# Snakebite Envenomation From the Large Palearctic Viper, *Macrovipera razii* (Squamata: Serpentes; Viperidae), in Fars Province, Southern Iran

**DOI:** 10.1155/jotm/4207010

**Published:** 2024-12-02

**Authors:** Saeed Shahabi, Kourosh Azizi, Aboozar Soltani, Azim Paksa, Mohammad Djaefar Moemenbellah-Fard, Mohsen Kalantari

**Affiliations:** Research Center for Health Sciences, Institute of Health, Department of Biology and Control of Disease Vectors, Shiraz University of Medical Science, Shiraz, Iran

**Keywords:** Abundance, envenomization, geographic distribution, Iran, snake

## Abstract

Snakebites are a significant health issue, especially in tropical and subtropical regions. Envenomation from snakebites is a clinical emergency requiring prompt treatment. Recently, a new species of blunt-nosed viper, *Macrovipera razii*, was identified in central and southern Iran through morphological and molecular studies. This large, dangerous viper can deliver substantial amounts of venom. Following reports to the Faculty of Health at Shiraz University of Medical Science (SUMS), the identification of venomous snakes involved in envenomation cases in Fars province was undertaken. Approximately 20 snakes were captured and presented by locals, while others provided photos. Despite some information being photo-based, the data highlighted the significant role of this viper in envenomation cases. *Macrovipera razii* is now recorded from 12 counties in Fars province. One incident involved a male bitten in Shiraz, and another case led to a male needing limb amputation. This study emphasizes the importance of this newly described viper in recent snakebite envenomations in the region and reviews its distribution within the Fars province.

## 1. Introduction

Snakebite is a significant medical emergency, leading many individuals to health centers each year. It can result in injuries, amputations, or even death. Thus, accurate knowledge and basic treatment methods are essential [1, 2]. Annually, there are about 5.5 million snakebites, 1.8 million cases of envenomation, and 94,000 deaths [1]. Most snakebites occur in South Asia, Southeast Asia, Sub-saharan Africa, and Latin America [2]. The majority of deaths from snakebites happen in Asia (estimated at 15,400–57,600 deaths per year) and Sub-Saharan Africa (3500–32,100 deaths per year) [3].

Snakes are categorized into three groups based on toxicity: venomous, semivenomous, and nonvenomous. Venomous snakes, primarily from the viperid and elapid families, are responsible for most snakebites, particularly in Iran [4]. Of the approximately 3000 identified snake species worldwide, only 83 species from four families are endemic to Iran [5]. Most elapids are venomous and found in tropical and subtropical regions, with many possessing neurotoxic venom delivered through hollow fangs containing various toxic components. Viperids have a pair of long, solenoglyphous fangs that can rotate and are used for venom injection. These fangs are located at the front of the mouth on a short maxillary bone and fold back against the roof of the mouth when not in use, encased in a membranous sheath [6].

In Iran, snakebite envenomation management strategies vary by center and are not standardized, often relying on the practical experiences from other countries. The venom composition of the same snake species can differ across regions due to environmental factors such as temperature and humidity. Additionally, the chemical ingredients of antivenoms may vary between countries. Therefore, it is unwise to apply the same treatment protocols that are effective elsewhere [7].

Snakebite envenomation is a clinically toxic emergency that requires prompt and organized treatment. An effective multifaceted protocol is essential to enhance clinical outcomes, minimize unnecessary antivenom use, and assist clinical judgment. In Iran, the management strategy for snakebite envenomation includes a severity grading scale, a schematic algorithm specific to snakebite cases, and guidelines for supportive treatments [8].

*Macrovipera*, the large Palearctic viper, includes two species in Iran, which account for numerous bites in Africa and Western Asia each year. Due to their size, these venomous snakes can inject a significant amount of venom, making them among the most dangerous. Recent morphological and molecular studies have confirmed the presence of two *Macrovipera* species in Iran [9]. The first, *Macrovipera lebetina*, has two subspecies (*obtusa* and *cernovi*) found in western, northwestern, and northeastern Iran [5]. The second, *Macrovipera razii*, named after the ancient Persian physician Abu-Bakr Zakariya Al-Rhazi, is a newly described species from central and southern Iran, including Fars province [9, 10]. This study aims to report the first case of snakebite envenomation by *M. razii* in Fars province and to highlight treatment strategies and preventive measures for bites from this dangerous viper.

## 2. Material and Methods

### 2.1. Study Area

**Figure 1:**
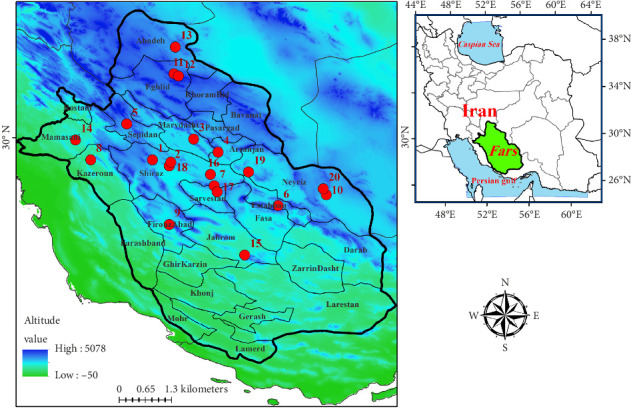
Distribution range of the viper *M. razii* in Fars province, southern Iran.

Fars province (29.1044°N, 53.0459°E), covering 1,22,400 km^2^, is located in southwestern Iran and is the fourth largest province after Sistan and Baluchistan, Kerman, and Yazd. The province features diverse landscapes, including mountainous regions and plains, with its mountains forming part of the extensive Zagros Mountain range, which runs northwest to southeast and connects to the southern coast. Fars experiences a variety of climates, influenced primarily by changes in altitude and latitude that affect temperature and precipitation [5]. This results in significant climatic differences across the province. In the southern and eastern regions, low rainfall and extreme heat lead to sparse vegetation, while saline and infertile soil limit agricultural potential. Most of the province's deserts, including the Larestan and Lamerd deserts, are found in these areas (Figure 1).

### 2.2. Snake and Snakebite Envenomation

Data on the distribution range of the viper *M. razii* were gathered from various sources, including literature [5, 9, 10], captured snakes by the fire brigade and the Department of Environment (DOE), and vipers killed by snakebite victims or their companions, along with two identifiable photographs. Live snakes were released back into the wild after identification, while killed specimens were preserved in formalin and stored at the Museum of Zoology and Entomology, Shiraz University of Medical Sciences (SUMS), for future educational and research use. *M. razii* can be morphologically distinguished from other vipers in Fars province [9]. Euthanizing of snakes was done using chloroform (CHCL3, Product No.: 208, CAS No.: 67-66-3, KIMIA EXIR CHEMICALS, Iran), but their brain manually destroyed [11]. Collecting and sending snakes in this project were done with the cooperation of environmental and firefighting organizations, and these organizations had the necessary permits to catch or release snakes in the environment, and the authors of the article only had the responsibility of identifying the sent snakes. Information and photographs of snakebite victims were collected with their full consent, ensuring anonymity. ArcMap software was used to create the distribution map. Of the approximately identified four viper species in southern Iran—*M. razii*, *Pseudocerastes persicus*, *Pseudocerastes fieldi*, and *Echis carinatus*—the studied viper, *M. razii* (previously known as *Macrovipera lebetina*), is easily distinguishable from others, even in photographs. However, we identified only two snakes from photos. The snakes responsible for the bites were identified using morphological characteristics in the field or museums. Recent studies indicate that the *Macrovipera* species in central and southern Iran are *M. razii*, not *M. lebetina*. We also consulted reptile experts for final confirmation of these findings [4, 9, 10].

## 3. Results

### 3.1. Species Distribution

**Figure 2:**
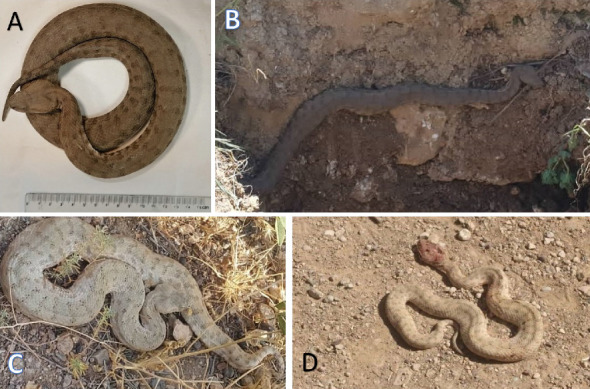
Original photos demonstrating the habitus of some specimens of M. razii identified in the present study from Shiraz (A), Sepidan (B), Estahban (C), and Sarvestan (D) regions. It shows the pear-shaped head and a series of darkly patched scales on its trunk.

**Figure 3:**
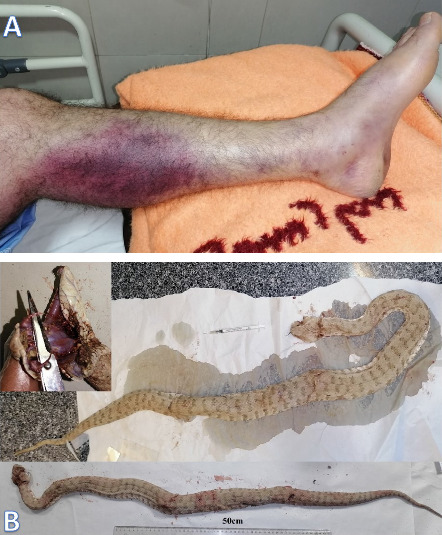
The left foot of a 46 years old (A) bitten by the snake *M. razii* (B) from mountainous area of Shiraz City (Derak). It shows a wide area of ecchymoses on the upper part of the calf muscle area of the patient. The long sharp fangs are discernible in the inlet of the lower photos.

In this study, 20 specimens of *M. razii* were identified in Shiraz (Saadi, Derak, and Bamoo National Park), as well as in Sepidan, Estahban, Kazerun, Sarvestan, Jahrom, and Firouzabad (Figures 2 and 3). According to published data, this species has also been reported in the following counties of Fars province: Neyriz (Ghatroyeh national park), Shiraz, Eghlid (Asopas), Eghlid (Tang-e-Karun), Abadeh, Mamasani, *Neyriz* (Bahram-e Gur) protected area, Arsanjan (Bakhtegan national park), *Shiraz* (Bamoo national park), Sarvestan, Kherameh (Seyf Abad) (5, 7). The distribution range of the viper, based on this study and existing data, is illustrated in Figure 1 and Table 1.

**Figure 4:**
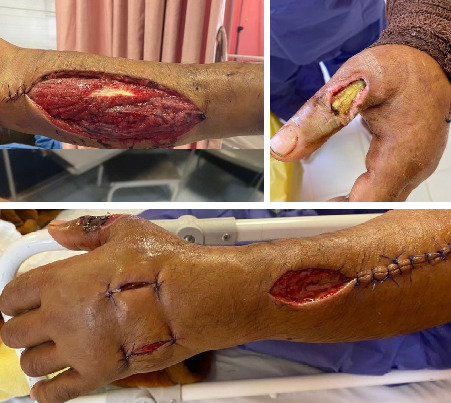
The hand of a 42 years old in Estahban County was bitten by a Levantine viper, *M. razii*. Due to extensive injuries, doctors ultimately had to amputate his hand.

In this study, two cases of snakebite envenomation were documented involving men over the age of 40.  Figure 3 depicts a man in Shiraz. The viper *M. razii* (Figure 3) was killed by the victim's companions and sent to SUMS, where it is now housed.  Figure 4 shows a man who was bitten in Estahban County (Figure 4). Unfortunately, tissue necrosis resulted in the amputation of part of the affected limb. In addition, there were undocumented reports in cyberspace of the death by this snake, as the observed image of the snake (Figure 2(D)) could confirm the bite by this viper.

### 3.2. The Treatment Strategies and Preventive Measures of Snakebite of *Macrovipera razii*

According to the recorded data, *M. razii* snakebites caused disturbances in the systemic hemodynamics of these patients, leading to reduced kidney function and serious symptoms such as edema, hypotensive shock, and tissue necrosis. Long-term musculoskeletal disabilities and swelling extended to the upper limbs within 24 h. Fasciotomy was performed on the affected areas of the upper limbs. Symptoms documented included severe localized pain and swelling, dizziness, weakness, low blood pressure, and itching around the snakebite site.

The recorded treatment protocol included hexavalent antisnake immunoglobulin, prepared from the purification and concentration of hypersafe horse plasma infused with venom from six dangerous Iranian snake species (purified, transparent, and injectable immunoglobulin with product code 3246).

The active components of this antidote included the F(ab')2 fragment of antisnake venom immunoglobulins, capable of neutralizing over 50 LD-50 of venom from each mentioned snake, based on its lethality in mice. It also contains a maximum of 0.25% phenol (w/v) as a preservative. This product was specifically prepared to neutralize snake venom and treat snakebite victims.

### 3.3. Dose, Method of Administration, and Route of Administration

Given the severity of symptoms and clinical complications, it was recommended to administer 1-2 ampoules intravenously as an initial dose to neutralize the snake venom. In cases of severe poisoning, especially with delays in treatment, a larger amount of antidote was necessary, determined by the attending physician based on test results and clinical examination. If coagulation issues persisted or recurred, the initial dose could be repeated after 6 h. Recorded side effects in these patients included dry cough, shortness of breath, urticaria, itching, nausea, vomiting, abdominal colic, diarrhea, hypotension, tachycardia, and anaphylactic shock. Delayed side effects, often caused by endotoxins and typically observed 1-2 h postadministration, included shivering, chills, fever, vasodilation, and hypotension.

Late side effects (serum sickness) appeared 1–12 days posttreatment, with an average onset around 7 days. Symptoms included fever, nausea, vomiting, diarrhea, itching, skin rash or hives, muscle pain, joint pain, and lymphadenopathy. Administration of antihistamines and corticosteroids reduced these late reactions. In the case of the second patient, a 42-year-old, the limb was amputated below the elbow due to worsening tissue necrosis. Ultimately, both patients were discharged from the hospital after about 2 weeks.

## 4. Discussion

The province of Fars features many mountainous regions that are part of the expansive Zagros Mountain range, which runs northwest to southeast. The distribution range of the viper *M. razii* (Figure 1) closely follows the Zagros Mountains in Fars. While this viper has been reported in some counties, it is likely to spread into the mountainous areas of other regions within the province. The city itself is a touristic destination in southern Iran, with its northern outskirts located along the foothills of the Zagros range. Climate changes, recent droughts, habitat destruction, and urbanization may affect the incidence of snakebites. Increased urbanization in the foothills, rising temperatures, food waste, and an increasing number of rodent populations can drive snakes toward buildings in Shiraz, leading to an increase in venomous snakebites and hospitalizations.

Given that biosystematic studies indicate *M. razii* differs from other viper species in Iran both molecularly and morphologically, there should be increased focus on research regarding its biological and medical aspects. This includes objectives such as proteomic identification and quantification of its venom, as well as studying the snake's ecology and behavior.

Two medically significant families of venomous snakes are Viperidae (vipers and pit vipers) and Elapidae (including kraits, mambas, cobras, and coral snakes). Other less important snakes, such as non-front-fanged species from Dipsadidae, Lamprophiidae, Natricidae, and Colubridae, rarely cause life-threatening envenoming [12]. Snake venom comprises proteins and peptides evolved to disrupt physiological pathways in prey, but they can severely affect humans when bitten. Clinical pathologies and pathophysiological effects of envenomation include neurotoxicity, hemotoxicity, and tissue damage, which encompass cytotoxicity, myotoxicity, and degradation of the extracellular matrix, with some venoms causing a combination of these effects. Neurotoxic effects involve toxins disrupting synaptic transmission by blocking specific ion channels, hydrolyzing phospholipids at the presynapse, or acting as antagonists at cholinergic receptors, ultimately impairing neuromuscular transmission. Some toxins lead to respiratory paralysis by inhibiting acetylcholine esterase in the synaptic cleft, causing excessive muscle stimulation, and resulting in fasciculation or spasms. Furthermore, α-neurotoxins and β-neurotoxins are responsible for neurotoxic effects, acting post- and presynoptically, respectively [13].

Although the injection of polyvalent antivenom is the first-line treatment for snakebite victims, morbidity and mortality rates remain high in many regions worldwide [14]. Many doctors struggle to identify venomous snake species or may not see the snake, leading to the frequent use of polyvalent antivenom, which can worsen patient complications compared to monovalent antivenom. Polyvalent antivenom contains antibodies against multiple snake species, neutralizing venom from a single bite [15, 16]. However, unnaturalized components pose additional health risks, highlighting the importance of accurate snake identification [12]. This study aims to increase awareness among doctors about the viper *M. razii*, which is endemic to central and southern Zagros and is responsible for the majority of venomous snakebites in Fars. Given the significance of this viper, it is recommended to develop more antivenom specific to it for health centers and hospitals.

In regions where venomous snakes are endemic, snakebite envenomation is a significant public health issue. Care facilities are crucial for managing severe cases. Improving awareness of treatment methods and outcomes associated with snakebites in these settings is essential for enhancing patient care and reducing mortality and morbidity rates [17].

Mortality rates and envenomation severity vary among snake species, with systemic symptoms often resulting in complications such as coagulopathy and respiratory failure [18]. Delayed treatment increases mortality and complication rates, while timely antivenom administration leads to better outcomes. Thus, early identification, prompt antivenom administration, and comprehensive supportive care—including antibiotics, treatments for coagulopathy, and analgesics—are crucial for patient recovery [19, 20].

## 5. Conclusions

This study highlights the significant role of the newly described viper species, *M. razii*, in recent snakebite envenomations in the region and reviews its distribution as an endemic species in Iran. A coordinated effort among various Iranian departments—such as health authorities, policymakers, public servants, antivenom manufacturers, the Natural Resources and Forestry Organization, and healthcare providers—could reduce snakebite morbidity and mortality rates. Some of these goals have already been achieved; mono- and polyvalent antivenoms for Viperidae, other medically significant snake families, and other poisonous animals including scorpions are now available in the country's hospitals and are used alongside other treatment methods [21–23].

## Figures and Tables

**Table 1:** Distribution range of the viper *M. razii* in Fars province, as determined by this study and existing data.

Number	Location	Latitude	Longitude
1	Shiraz, Derak	29.714394	52.394119
2	Shiraz, Sadi	29.63266	52.605949
3	Shiraz, Bamoo national park	29.98	52.93
4	Arsanjan, ZiadAbad	29.808631	53.248854
5	Sepidan	30.17775	52.051115
6	Estahban	29.109716	54.038718
7	Sarvestan	29.365659	53.195024
8	Kazeroun	29.714864	51.580007
9	FirouzAbad	28.862805	52.612436
10	Neyriz (Ghatroyeh national park)	29.247494	54.668071
11	Eghlid (Aso pas)	30.839996	52.66741
12	Eghlid (Tang-e-Karun)	30.8095	52.730493
13	Abadeh	31.194381	52.692151
14	Mamasani	29.97014	51.3776
15	Jahrom, Gorm protected area	28.456198	53.600068
16	Kherameh (Seyf Abad)	29.5184	53.150846
17	Sarvestan	29.288687	53.239328
18	Shiraz, Bamoo national park	29.68	52.63
19	Arsanjan, Bakhtegan national park	29.55	53.65
20	Neyriz (Bahram-e Gur protected area)	29.33	54.63

## Data Availability

The data supporting the findings of this study are available within the article.
